# Effectiveness Index of Mechanical Energy Utilization in Male 400‐m Sprinters and the Relation Between Muscle Cross‐Sectional Area of the Trunk and the Lower Limb

**DOI:** 10.1111/sms.70023

**Published:** 2025-02-08

**Authors:** Kotaro Muratomi, Takashi Tarumi, Yuki Furuhashi, Naoki Ushirooka, Daisuke Hoshi, Marina Fukuie, Takahiro Hasegawa, Jun Sugawara, Satoru Tanigawa, Hideyuki Takahashi, Hirohiko Maemura

**Affiliations:** ^1^ Graduate School of Comprehensive Human Sciences University of Tsukuba Ibaraki Japan; ^2^ Human Informatics and Interaction Research Institute National Institute of Advanced Industrial Science and Technology Ibaraki Japan; ^3^ Japan Society for the Promotion of Science Tokyo Japan; ^4^ Japan Overseas Cooperation Volunteers Tokyo Japan; ^5^ Faculty of Health and Sports Science University of Tsukuba Ibaraki Japan

**Keywords:** economical sprinting, long sprinter, magnetic resonance imaging, mechanical work, morphological characteristics

## Abstract

The purpose of this study was to identify the morphological characteristics of trunk and lower limb muscles associated with 400‐m sprint performance and “effectiveness index of mechanical energy utilization (EI).” Twelve male 400‐m sprinters (age: 21.4 ± 1.7 years, 400‐m best time: 48.38 ± 1.80 s) participated in this study. Using a 3‐T magnetic resonance imaging system, we measured muscle cross‐sectional area (CSA) of the key trunk and lower limb muscles, including the rectus abdominis, lateral abdominal wall, erector spinae, psoas major, adductors, quadriceps femoris, hamstrings, dorsiflexors, and plantar flexors. The CSA of the trunk muscles was summed bilaterally, while the CSA of the lower limb muscles was assessed on the right leg. In addition to absolute CSA, relative CSA, normalized by the two‐thirds power of lean body mass, was used in the analysis to account for individual body size differences. Participants completed a 400‐m sprint on an official outdoor track, with running motion in early phase (around the 160‐m point) analyzed using high‐speed video cameras. Our findings indicate that the specific muscle groups' relative CSA, particularly the lateral abdominal wall and adductors, were significantly correlated with 400‐m sprint time (*r* = −0.604 to −0.748, *p* = 0.005–0.038) and EI (*r* = 0.598–0.599, *p* = 0.040). Additionally, the thigh‐to‐lower leg CSA ratio was significantly correlated with 400‐m sprint time (*r* = −0.643, *p* = 0.024) and EI (*r* = 0.577, *p* = 0.049). These results suggest that the relative size of proximal muscles plays a crucial role in economical sprinting in 400‐m sprinters. Thus, optimizing the development of proximal muscles relative to distal muscles may enhance 400‐m sprint economy, providing a valuable reference for designing training programs focused on economical running techniques.

## 1 Introduction

Lower body muscle strength is considered important for better sprinting performance [1]. Muscle strength is largely determined by the size of the muscle cross‐sectional area (CSA) [2, 3]. The CSA of many muscles in the trunk and lower limbs is greater in short sprinters than in non‐sprinters [4, 5]. In addition, the absolute CSA of several muscle groups such as the psoas major, hamstring, and adductors is related to short sprint performance [6, 7, 8]. Thus, the larger CSA of trunk and thigh muscles appears to be a key morphological characteristic of elite short sprinters. However, most of the previous studies examined the relation between muscle CSA and “short” sprint performance (e.g., 30–100 m); thus, the relation with “long” sprint performance (e.g., 400‐m) remains poorly understood.

In contrast to short sprints, the 400‐m sprint requires the ability to attain maximal sprint velocity and maintain it over a longer duration [9]. Thus, 400‐m sprinters require economical sprinting techniques to maintain high velocity with minimal energy expenditure [10, 11]. Previous studies have reported that “large” muscles in the lower limbs determine explosive muscle strength [2, 3], whereas “too large” muscles in the lower limbs lead to an increase in the moment of inertia and thus energy expenditure [12, 13]. This trade‐off underscores the necessity of distinct training adaptations: Whereas short sprinters may benefit from larger muscles for explosive power, 400‐m sprinters may gain more advantage by optimizing the distribution and size of their muscles to balance power generation with efficient energy expenditure. Indeed, short sprinters have shown that thigh muscle CSA is correlated with short sprint performance [7, 14], whereas long‐distance runners, who emphasize running economy, do not exhibit such a relation and tend to have smaller lower leg muscles [15]. This muscle morphology found in long‐distance runners enables an efficient power output by having a low moment of inertia and thus requiring less muscular effort in leg swing [16]. Based on these findings, a greater proportion of proximal compared to distal muscle mass may positively contribute to 400 m performance by enabling more economical sprinting techniques while maintaining sufficient power. Therefore, it can be hypothesized that 400‐m sprint performance may be related not to the actual size of the muscle itself (i.e., absolute size), but rather to the size of the muscle in relation to overall body mass or in comparison to other muscles (i.e., relative size).

In our previous study, we quantified economical techniques in 400‐m sprinters as “effectiveness index of mechanical energy utilization (EI)” which was defined as “sprinting with a longer stride length with less mechanical work” and calculated as stride length divided by whole body mechanical work. A higher EI indicates the ability to achieve a larger stride with less mechanical work. We found that elite 400‐m sprinters have a high EI in the early phase of the race [17]. However, the morphological characteristics involved in economical techniques (i.e., EI) of 400‐m sprinters have not been investigated in detail. Identifying the muscle CSA associated with economical techniques can further provide new insights into training interventions to improve competitive performance in the 400‐m sprint. Therefore, the purpose of this study was to identify the morphological characteristics of the trunk and lower limb muscles associated with 400‐m sprint performance and EI, focusing on muscle CSA. We hypothesized that EI is not related to the absolute muscle CSA but to the relative CSA.

## 2 Materials and Methods

### 2.1 Participants

Twelve male sprinters (age: 21.4 ± 1.7 years) participated in this study. Participants had specialized and competed regularly in the 400‐m race. Their personal best times in the 400‐m race ranged from 46.60 to 52.67 s (mean 48.38 ± 1.80 s). An a priori power analysis with an assumed type 1 error of 0.05 and a type 2 error rate of 0.20 (80% statistical power), *r* = 0.70 (strong correlation) [18] was conducted for each variable. The results showed that 12 participants would be sufficient to find the statistically significant correlation between “performance and muscle CSA.” The inclusion criterion was specialization and regular competition in the 400‐m race. The exclusion criteria were as follows: use of medications affecting exercise capacity or having orthopedic limitations. All participants were fully informed of the purpose, methods, and risks of the study, and their consent to participate in the experiment was obtained. This experimental procedure was approved by the Human Research Ethics Committee at the University of Tsukuba (approval number: PE022‐37) and the Institutional Review Board of the National Institute of Advanced Industrial Science and Technology and was performed by the guidelines of the Declaration of Helsinki and Belmont Report.

### 2.2 Magnetic Resonance Imaging

**FIGURE 1:**
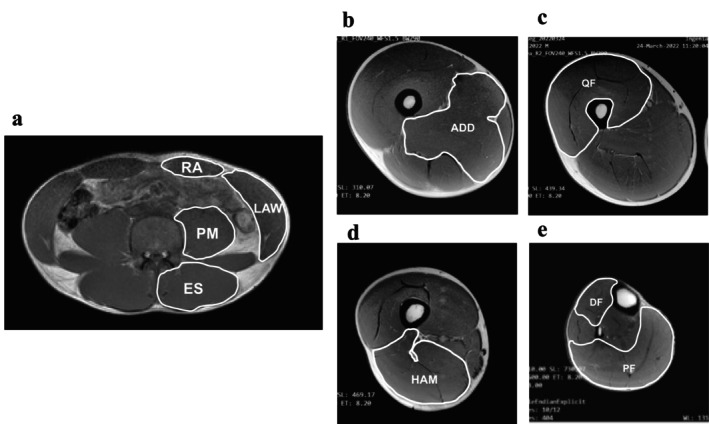
Representative magnetic resonance imaging scans for measuring cross‐sectional areas (CSA) of trunk and lower limb muscles. These show the mid‐level of L4–L5 (a), the proximal 30% (b), 50% (c), and 70% (d) of the thigh length, and the proximal 30% of the lower leg length (e). The cross‐sectional areas of the rectus abdominis (RA), lateral abdominal wall (LAW), elector spinae (ES), psoas major (PM), the adductors (ADD), quadriceps femoris (QF), hamstrings (HAM), dorsiflexors (DF), and plantar flexors (PF) were obtained. The thigh‐to‐lower leg CSA ratio is the ratio of the CSA of all muscles in the proximal 30% of the thigh (b) to that of the lower leg (e).

Representative magnetic resonance imaging (MRI) images for measuring the CSA of the trunk and lower limb muscles are shown in Figure 1. MRI measurement was taken using a 3‐T system (Ingenia, Philips Medical Systems, The Netherlands). Participants were scanned in the supine resting condition on the scanner bed. A cushion was placed under the buttocks and knees to avoid morphological change and full knee extension. Feet were placed in a custom‐made platform to avoid movement during imaging. After the acquisition of the localizer image, transverse T1‐weighted images were collected to measure trunk muscles with the following parameters: field of view (FOV) = 380 × 380 mm^2^, matrix = 512 × 512 pixels, in‐plane resolution = 1.4 × 1.4 mm^2^, slice thickness = 10 mm, 11 slices (gap = 2 mm), and scan duration = 21.8 s. The center of the FOV was placed at the level of L4–L5 confirmed by the sagittal localizer image. Trunk images were taken during breath‐holding to reduce the effect of respiratory motion. Next, the right lower limb muscles were measured by transverse T1‐weighted imaging with the following parameters: FOV = 240 × 240 mm^2^, matrix = 512 × 512 pixels, in‐plane resolution = 1 × 1 mm^2^, slice thickness = 10 mm, 12 slices (gap = 5 mm), and scan duration = 1 min 22 s per scan. Five imaging stacks were collected to cover the whole limb starting from the top of the greater trochanter to the bottom of the foot.

One examiner blinded to participant characteristics manually traced the trunk and lower limb muscles. Regarding the trunk muscles, the CSA of the rectus abdominis, lateral abdominal wall, erector spinae, and psoas major were obtained at the mid‐level of the L4–L5 (L: lumbar spine) [7, 19]. The CSA of the lateral abdominal wall included the transverse oblique, internal oblique, and external oblique. With regard to the lower limb muscles, the CSA of the adductors, quadriceps femoris, hamstring, dorsiflexors, and plantar flexors were measured [8, 20]. The images for calculating the CSA of the lower limb muscles were obtained based on methods described in previous studies [21, 22, 23]. In other words, the adductors' CSA was obtained at the proximal 30% of the thigh length (i.e., the distance between the greater trochanter and the lower edge of the femur). The quadriceps femoris CSA was obtained at the proximal 50% of the thigh length. The hamstring's CSA was obtained at the proximal 70% of the thigh length. The dorsiflexors and plantar flexors' CSA were obtained at the proximal 30% of the lower leg length (i.e., the distance between the superior end of the tibia and the lateral malleolus of fibula). The thigh‐to‐lower leg CSA ratio was calculated as the ratio of the CSA of all muscles in the proximal 30% of the thigh to that of the lower leg. The CSA of trunk muscles was summed bilaterally, and the CSA of lower limb muscles was measured in the right leg. Image analysis software (ImageJ, NIH, Bethesda, MD, USA) was used to measure CSA. In addition to absolute CSA, relative CSA normalized by the two‐thirds power of lean body mass was used in the analysis to minimize the effect of body size differences among participants (Equation 1) [24]. To assess the reliability of manual tracing, all muscles were traced twice.

(1) $$\text{Relative}\;\mathrm{CSA}=\frac{\text{Absolute}\;\mathrm{CSA}}{\text{Lean}\;{\text{body mass}}^{\frac{2}{3}}}$$

### 2.3 400‐m Sprint Test

**FIGURE 2:**
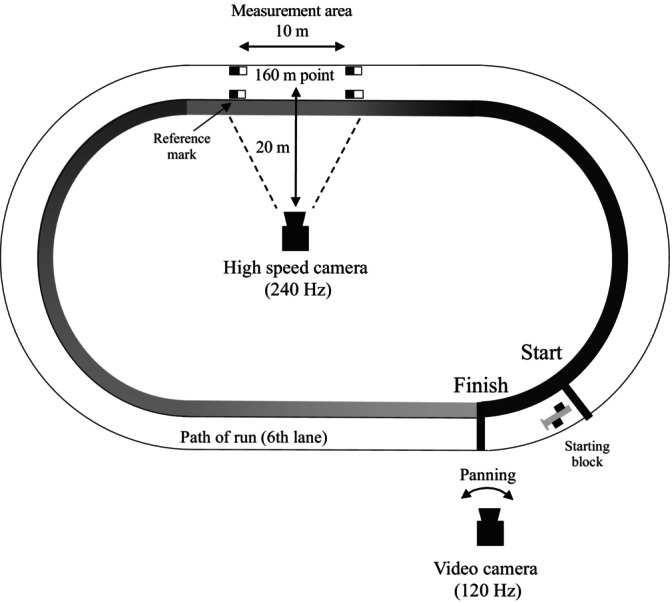
Set‐up for the 400‐m sprint test. The sprint test was performed on the sixth lane of an official outdoor 400‐m track, using the starting block. The entire race was recorded using a panning camera at 120 Hz. Additionally, sagittal plane motion around the 160‐m point was captured using a high‐speed camera at 240 Hz.

The set‐up for the 400‐m sprint test is shown in Figure 2. Participants performed the 400‐m sprint test in their own competition spiked shoes after a self‐selected warm‐up. The sprint test was conducted on the sixth lane of an official outdoor 400‐m track, using starting blocks. Participants were instructed to run as if they were in a race and were not given any instructions regarding pacing tactics. The experiment was conducted during the competitive season to reflect the participants' performance under typical race conditions. One panning camera (HC‐VX992MS, Panasonic, Osaka, Japan, frame rate: 120 fps) was used to calculate the 400‐m sprint time from the side of the finish line. Additionally, one fixed camera (LUMIX DC‐GH5S; Panasonic, Osaka, Japan, frame rate: 240 fps) was used to analyze the sagittal plane running motion around the 160‐m point from the starting line [17, 25].

**FIGURE 3:**
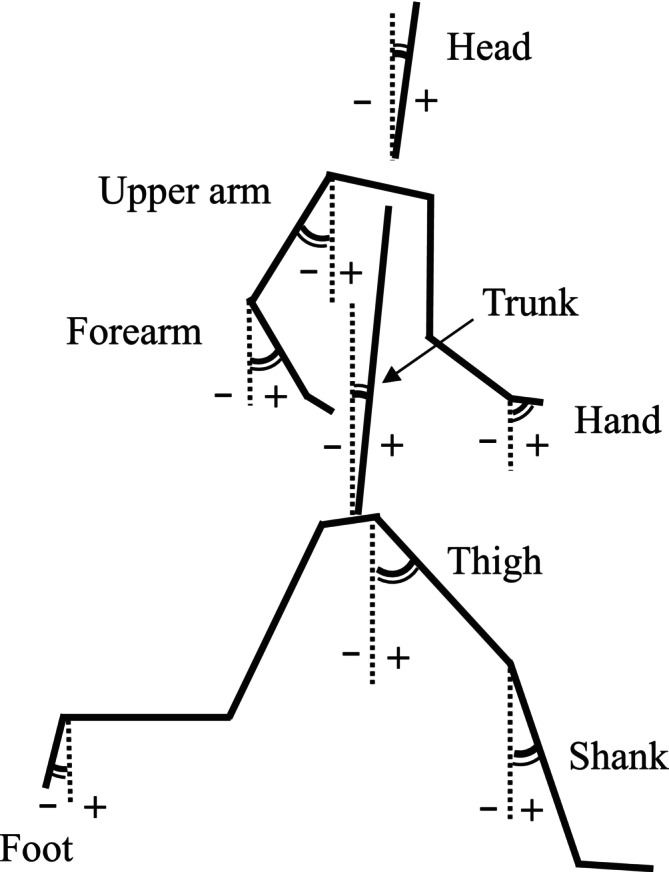
Definitions of the segment angles.

The analyses for measuring 400‐m sprint time were conducted using QuickTime Player (version 7; Apple, USA). Based on the flash of the pistol, the frames were examined at the moment the torso crossed the finish line. The difference between these frames was used to calculate the time of the 400‐m sprint time. Two‐dimensional coordinates of 23 body landmarks were obtained using the video digitizing system (Frame‐DIAS; DKH, Tokyo, Japan). These coordinates were smoothed using a fourth‐order zero‐phase shift Butterworth low‐pass digital filter with a cut‐off frequency of 6 Hz, as derived from the residual analysis [26]. The instantaneous displacement and velocity of the center of mass were calculated frame‐by‐frame over one stride cycle, defined as the period from the initial contact of the left foot to the subsequent initial contact of the same foot. The velocity was calculated as the resultant of the horizontal and vertical components of the center of mass in the sagittal plane. The angular velocity of each segment was determined using instantaneous frame‐by‐frame calculations in the sagittal plane (Figure 3). The center of mass was calculated according to a previous study on Japanese athletes [27].

The EI calculation procedure is described below. First, the instantaneous total mechanical energy (*E*_*i*,*j*_) of the “*i*th” segment at time instant “*j*” (recorded at 1/240 s intervals) was calculated using Equation (2) [28, 29].

(2) $$E_{i,j}=m_{i}{\mathrm{gh}}_{i,j}+\frac{1}{2}m_{i}v_{i,j}^{2}+\frac{1}{2}I_{i}\omega_{i,\;j}^{2}$$

where “*m*_*i*_” is the segment mass, “*g*” is the acceleration of gravity, “*h*_*i*,*j*_” is the height of the segment center of mass, “*v*_*i*,*j*_” is the instantaneous velocity of the segment center of mass, “*I*_*i*_” is the moment of inertia around the segment center of mass, and “*ω*_*i*,*j*_” is the instantaneous angular velocity of the segment. Equation (3) was then used to determine the difference in the mechanical energy of the segment between consecutive time instants, which was taken as the change in the mechanical energy of the segment.

(3) $$∆E_{\mathrm{ij}}=E_{\mathrm{ij}+1}-E_{\mathrm{ij}}$$

The mechanical work (*W*_wb_) was calculated from the mechanical energy, assuming energy transfer within and between segments using the approach of Pierrynowski et al. (Equation 4) [29, 30].

(4) $$W_{\mathrm{wb}}=\sum_{j=1}^{n-1}\left|\sum_{i=1}^{s}\left(∆E_{\mathrm{ij}}\right)\right|$$

where “*n*” is the number of frames per one stride, and “*s*” is the number of segments, which is 14 in this study.

To evaluate whether the mechanical energy was effectively utilized for the horizontal movement of the body, EI was calculated using Equation (5) [17].

(5) $$\mathrm{EI}=\frac{d}{W_{\mathrm{wb}}}$$

where “*d*” is the distance the body's center of mass moves horizontally during one stride and “*W*_wb_” is the mechanical work done by the body during one stride. A larger EI means that a longer stride length is achieved with less mechanical work, an important mechanical variable for 400‐m sprinters [17].

### 2.4 Statistical Analysis

For muscle CSA, the mean data of the two traces were used as representative values for each participant. The intraclass correlation coefficient (ICC) was used to assess the intraclass reliability of the muscle CSA obtained in the examiner's traces [31]. The Shapiro–Wilk test was used to check the normality of the distribution of the analyzed variables. The results confirmed normality for > 95% of the variables. The relation between muscle CSA and 400‐m sprint performance was evaluated using Pearson's product–moment correlation coefficient (*r*). The correlation coefficient was classified as negligible (0.00–0.10), weak (0.10–0.39), moderate (0.40–0.69), strong (0.70–0.89), and very strong (0.90–1.00) [18]. The significance level for all tests was set at *α* = 0.05. All statistical analyses were performed using SPSS software (version 29; IBM, Armonk, NY, USA).

## 3 Results

**FIGURE 4:**
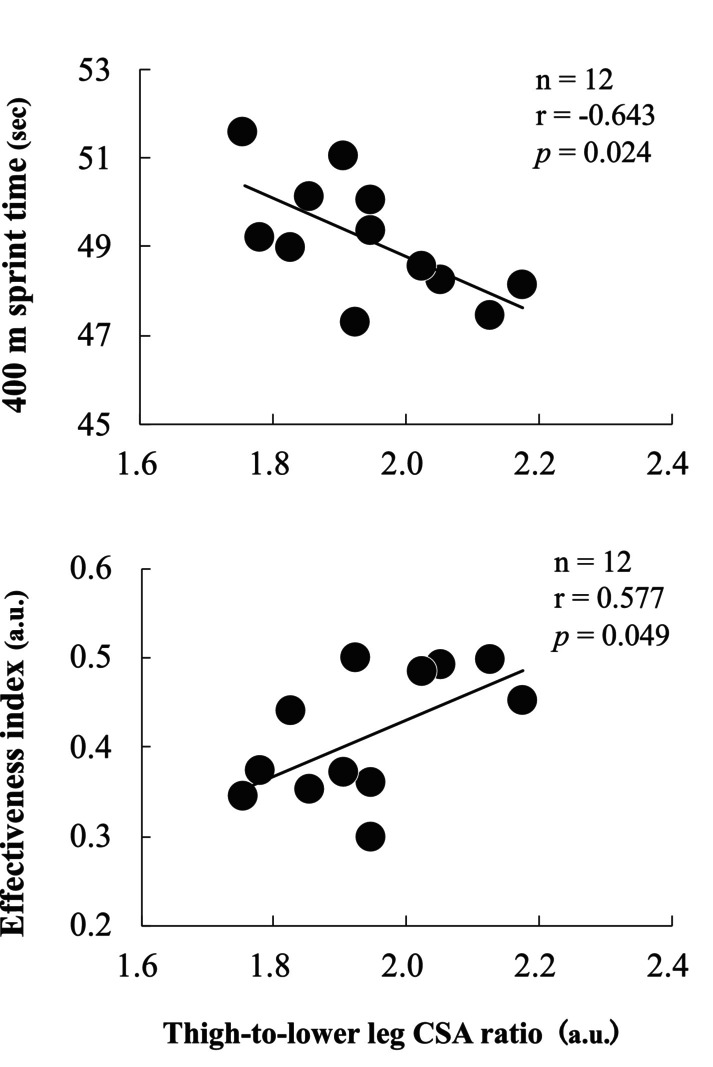
Correlation between thigh‐to‐lower leg CSA ratio at the proximal 30% and 400‐m running time, effectiveness index.

**FIGURE 5:**
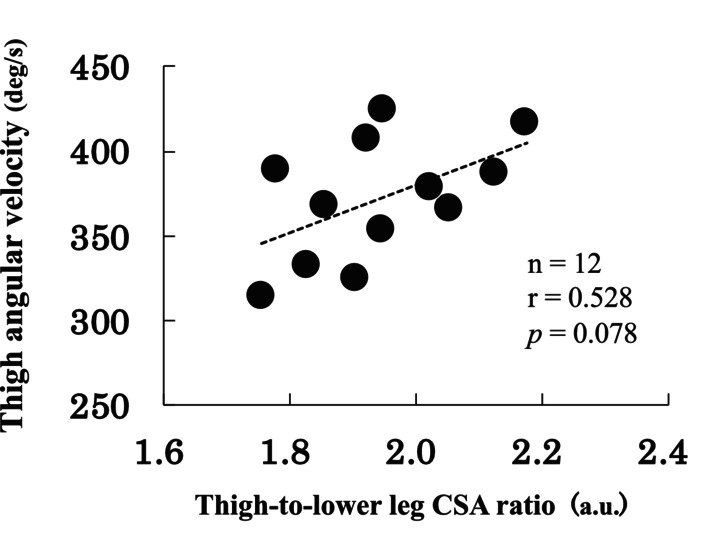
Correlation between thigh‐to‐lower leg CSA ratio at the proximal 30% and the average thigh angular velocity during the entire gait cycle.

Physical characteristics, the 400‐m sprint test, and the absolute and relative CSA of the trunk and lower limb muscles are shown in Table 1. Excellent reliability (ICC (1, 2) > 0.989, *p* < 0.001) was confirmed for all measured muscle CSA values. The relations between the 400‐m sprint test and CSA are listed in Table 2. Regarding the trunk muscles, the absolute and relative CSA of the lateral abdominal wall showed a significant moderate correlation with the 400‐m sprint time (*r* = −0.604 to −0.628, *p* = 0.029–0.038). The relative CSA of the lateral abdominal wall also showed a significant moderate correlation with EI (*r* = 0.599, *p* = 0.040). With regard to the lower limb muscles, the absolute CSA of the plantar flexors showed a significant moderate correlation with EI (*r* = −0.598, *p* = 0.040). However, no significant correlations were found between the absolute CSA of other lower limb muscles and EI. Contrastingly, in terms of relative CSA to lean body mass, the relative CSA of the adductors showed significant moderate to strong correlations with the 400‐m sprint time (*r* = −0.748, *p* = 0.005) and EI (*r* = 0.598, *p* = 0.040). The thigh‐to‐lower leg CSA ratio also showed significant moderate correlations with 400‐m sprint time (*r* = −0.643, *p* = 0.024) and EI (*r* = 0.577, *p* = 0.049) (Figure 4). Additionally, the thigh‐to‐lower leg CSA ratio showed moderate but non‐significant correlations with the average thigh angular velocity during the entire gait cycle (*r* = 0.528, *p* = 0.078) (Figure 5).

**TABLE 1:** Mean values of physical characteristics and absolute and relative cross‐sectional areas in 400‐m sprinters.

	Mean ± SD	Range
Height, cm	177.2 ± 14.7	170.4–189.0
Mass, kg	67.3 ± 5.3	57.1–76.6
Lean body mass, kg	61.6 ± 4.6	51.2–68.2
400‐m sprint time, s	49.1 ± 1.3	47.3–51.5
EI^a^	0.41 ± 0.07	0.30‐0.50
**Absolute CSA** ^**b**^, **cm** ^**2**^		
Rectus abdominis	17.6 ± 2.3	13.0–20.1
Lateral abdominal wall	56.7 ± 4.0	50.6–62.2
Erector spinae	54.7 ± 7.3	42.4–65.6
Psoas major	45.1 ± 6.4	31.4–52.8
Quadriceps femoris	85.2 ± 6.1	72.7–93.2
Hamstring	45.5 ± 6.1	38.3–56.4
Adductors	68.6 ± 7.0	60.2–84.7
Dorsiflexors	11.0 ± 1.5	9.3–14.1
Plantar flexors	53.1 ± 6.4	43.1–63.3
**Relative CSA** ^**b**^, **cm** ^**2**^ **/kg** ^**2/3**^		
Rectus abdominis	1.13 ± 0.13	0.88–1.32
Lateral abdominal wall	3.65 ± 0.33	3.17–4.06
Erector spinae	3.51 ± 0.41	2.90–4.17
Psoas major	2.90 ± 0.38	1.99–3.34
Quadriceps femoris	5.47 ± 0.25	5.07–5.81
Hamstring	2.91 ± 0.32	2.39–3.48
Adductors	4.40 ± 0.35	3.91–5.08
Dorsiflexors	0.70 ± 0.07	0.61–0.87
Plantar flexors	3.40 ± 0.31	3.08–3.89

^a^ Effectiveness index of mechanical energy utilization.

^b^ Cross‐sectional area.

**TABLE 2:** Correlation coefficients between the cross‐sectional area of trunk and lower limb muscles and 400‐m running time, effectiveness index.

	400‐m running time		EI^a^	
	*r*	*p*	*r*	*p*
**Absolute CSA** ^**b**^				
Rectus abdominis	−0.171	0.596	0.060	0.854
Lateral abdominalwall	−0.628	0.029	0.561	0.058
Erector spinae	−0.092	0.777	−0.112	0.729
Psoas major	−0.445	0.147	0.180	0.576
Quadriceps femoris	0.143	0.657	−0.293	0.356
Hamstring	0.079	0.807	−0.267	0.401
Adductors	−0.489	0.107	0.332	0.292
Dorsiflexors	0.289	0.362	−0.124	0.700
Plantar flexors	0.439	0.153	−0.598	0.040
**Relative CSA** ^**b**^				
Rectus abdominis	−0.278	0.381	0.192	0.550
Lateral abdominal wall	−0.604	0.038	0.599	0.040
Erector spinae	−0.176	0.583	−0.029	0.929
Psoas major	−0.563	0.056	0.304	0.337
Quadriceps femoris	−0.026	0.935	−0.131	0.685
Hamstring	0.015	0.963	−0.202	0.530
Adductors	−0.748	0.005	0.598	0.040
Dorsiflexors	0.254	0.426	−0.021	0.948
Plantar flexors	0.447	0.145	−0.628	0.029

^a^ Effectiveness index of mechanical energy utilization.

^b^ Cross‐sectional area.

## 4 Discussion

In this study, we investigated the morphological characteristics of the trunk and lower limb muscles associated with 400‐m sprint performance and EI. The economical sprinting techniques in 400‐m sprinters were defined as “sprinting with a longer stride length with less mechanical work” and this was named “the effectiveness index of mechanical energy utilization (EI).” [17] The present study showed that economical sprinters (i.e., sprinters with high EI in the early phase of the 400‐m race) do not necessarily have large absolute CSA of the lower limb muscles. Rather, a larger absolute CSA of the plantar flexors was associated with a lower EI. Meanwhile, there were significant positive correlations between the relative CSA of the lateral abdominal wall, adductors, and EI. Additionally, the larger the CSA of the thigh relative to the lower leg was associated with higher EI. Thus, the findings of the present study suggest that the economical sprinting in 400‐m sprinters is most likely related to relative rather than absolute muscle size. To the best of our knowledge, this is the first study to show a relation between economical techniques and muscle CSA in 400‐m sprinters.

The trunk cannot move quickly due to its large mass and moment of inertia because moving the trunk requires a large amount of mechanical energy. Therefore, when running with less energy, it is more economical to move the trunk as little as possible [32]. Deep trunk muscles, such as the transversus abdominis and internal obliques, stabilize the trunk by controlling intra‐abdominal pressure [33, 34] and maintaining optimal pelvic positioning during running [35]. The external obliques are also activated in coordination with lumbar pelvic movements as the running speed increases, controlling excessive trunk rotation and anterior pelvic tilt, and assisting in the forward swing of the legs [36, 37]. Thus, the larger CSA of the lateral abdominal wall of the elite 400‐m sprinters in the present study seems to be the result of these muscles acting repeatedly during the sprint to stabilize the trunk and assist the swing of the legs. In the present study, the transverse oblique, internal oblique, and external oblique were measured together as the lateral abdominal wall, so the extent to which each muscle is affected is unknown. However, the activity of these muscles seems to be strongly involved in the economical sprinting of the 400‐m sprinters. In contrast, we found no significant correlation between the CSA of the psoas major and 400‐m sprint time or EI. The psoas major is mainly responsible for hip flexion and is strongly related to sprint performance, especially stride frequency [20, 38]. Sprinters in 400‐m races need to compromise stride frequency to control fatigue and achieve a longer stride length throughout the race [39]. Thus, the non‐significant correlations between CSA of the psoas major and 400‐m sprint time and EI may be due to the fact that it is not as important for 400‐m sprinters as it is for 100‐m sprinters, who require a higher stride frequency.

Regarding lower limb muscles, we found no significant correlation between absolute CSA and 400‐m sprint time or EI for any muscle except the plantar flexors. These results were consistent with our hypothesis. In general, absolute CSA is related to maximal strength [2, 3], and therefore, larger absolute CSA in the lower limb muscles (e.g., the hamstring and adductors) is considered advantageous for short sprinters [6, 7, 8]. In contrast to the short sprinter, 400‐m sprinters need to reduce energy expenditure to control fatigue during the race [9, 10, 11]. If the lower limb muscle mass is too large, it increases energy expenditure during running, especially during leg swinging [12, 13]. Thus, these results may explain why there is a significant correlation between absolute CSA of the lower limb muscles and 100‐m sprint time but not 400‐m sprint time. Additionally, the present study showed a significant negative correlation between CSA and EI of the plantar flexors. This finding suggests that a large CSA of the plantar flexors has a negative effect on economical sprinting. Tottori et al. [8] showed that although the absolute CSA of the plantar flexors was larger in short sprinters compared to non‐sprinters, this did not correlate with personal best 100 m sprint time within the sprinter group. Multiple studies have consistently found no significant correlation between the CSA or muscle size of the plantar flexors and sprint performance in elite sprinters [40, 41]. Meanwhile, in long‐distance runners, large lower leg size appears to reduce running economy [15, 16]. A smaller lower leg muscle size may result in better long‐distance running performance by having a low moment of inertia and thus requiring less muscular effort in leg swing [16, 42, 43]. Thus, the result for the relation between the size of plantar flexors and 400‐m performance supports previous findings in long‐distance runners.

We also found a significant correlation between relative CSA of the adductors, 400‐m sprint time, and EI in terms of size relative to total body muscle mass. Adductors are known to be involved not only in hip adduction but also in hip extension to flexion and flexion to extension, depending on the lower limb position. In other words, the quantitative development of the adductors influences the exertion of force and torque during the thigh swing [44]. The generation of joint forces and torques contributes to the energy flow between the segments [45]. The more energy flow, the unnecessary mechanical work is minimized, leading to a higher EI [17]. The positive correlation between relative CSA and EI suggests that this muscle plays an important role in optimizing energy transfer and the effective utilization of mechanical energy. Additionally, adductors have a maximal CSA more proximal than other thigh muscles (e.g., quadriceps and hamstrings) [21, 22, 23]. This proximal distribution likely helps control the increase in lower limb moment of inertia caused by muscle hypertrophy. Thus, the large proportion of adductors relative to total body muscles may be related to efficiently generating power.

There were significant correlations between the thigh‐to‐lower leg CSA ratio and 400‐m performance and EI (Figure 4). In brief, elite 400‐m sprinters have a smaller CSA in the lower leg relative to the thigh. This finding supports that the lower limb showed different morphological development proximally and distally, and the development of relatively large proximal muscles was important to improve sprint performance [40]. In general, larger muscle size, reflected by an increased CSA, enhances the muscle's ability to generate force and perform work [2, 3]. However, this increase in muscle size also leads to greater mass and moment of inertia, which can require more work to move the segment. Interestingly, when a greater proportion of muscle mass is distributed proximally rather than distally, this additional demand for work may be mitigated [46]. This implies that even with the same total muscle mass, proximal muscle distribution reduces the effort required for segmental motion. The findings of our study, which showed that a higher thigh‐to‐lower leg CSA ratio was associated with a higher EI, align with this perspective. Although no statistically significant correlation was found, a trend was observed indicating a positive relation between the thigh‐to‐lower leg CSA ratio and the average thigh angular velocity during the entire gait cycle (Figure 5). This trend may suggest that a greater thigh‐to‐lower leg CSA ratio contributes to 400‐m sprint performance by facilitating efficient thigh motion. A previous study has reported that sprinting techniques emphasizing the forward swing of the thigh are associated with higher EI by enhancing energy flow between segments [17]. Additionally, smaller distal limb segments, as observed in long‐distance runners, reduce the moment of inertia and minimize the muscular effort required for leg swing, contributing positively to running economy [16]. Although no statistically significant correlation was found in the present study, these findings provide a framework for considering the importance of the distribution of muscle mass between the proximal and distal regions in improving 400‐m sprint performance.

The present study has some limitations. First, the study was conducted only in Japanese males, and therefore, the results of this study may not be directly applicable to other races or to females. Future research could benefit from exploring these relations in female athletes and in other racial groups to determine whether similar trends are observed across different populations. Second, we calculated the EI on the basis of whole body mechanical work, and therefore, the mechanical work of each segment is unknown. Additionally, since the running motion, which was originally performed in three dimensions, was explained using two‐dimensional variables, errors may have occurred. Future analyses should be carried out for each body segment in three dimensions to investigate the relation between each muscle CSA and mechanical energy generation in more detail. Third, although we used the CSA for evaluating muscle size, it has been considered that muscle volume is a more reliable marker of muscle size than CSA. The CSA of the lower limb muscles was also measured on the right side only and therefore does not take into account left–right differences in muscle size. Especially, differences in kinesiology between the left and right lower limbs during curve sprinting may result in inter‐limb variations in muscle CSA. Further studies focusing on muscle volume and left–right differences in muscle are needed to further clarify the findings of the present study. Fourth, this study did not control for pacing tactics or fatigue, which may have influenced the observed results. Future studies should incorporate methods to control these factors to better understand their impact on sprint performance. Fifth, correlation analysis cannot determine causality. As the participants in this study routinely performed strength training with muscle hypertrophy, it cannot be known whether the muscle CSA was determined by strength training or by training with running. Therefore, it is not clear whether a more economical form of sprinting led to a muscle morphology with a smaller moment of inertia or whether a muscle morphology led to a smaller moment of inertia and improved the economy of sprinting. Finally, the present study only examined the specific discipline of the 400‐m sprint. Future research should more extensively investigate the relation between muscle size and performance in different events, such as middle‐distance running and track cycling. However, this study provides new insights that elite 400‐m sprinters have greater muscle CSA in their thighs relative to their lower legs.

## 5 Perspectives

The findings of this study suggest that a large lateral abdominal wall is associated with economical sprinting in 400‐m sprinters. In contrast, elite 400‐m sprinters do not necessarily have a large absolute CSA of lower limb muscles. Conversely, larger plantar flexors may negatively impact economical sprinting. Additionally, the size of the thigh relative to the lower leg and adductors relative to total body muscle mass were associated with better 400‐m sprint performance. Therefore, increasing proximal muscles relative to the distal muscles can lead to more economical sprinting. Based on these results, we recommend that coaches focus on strengthening specific muscle groups, particularly the lateral abdominal wall and adductors, rather than aiming for general hypertrophy of all lower limb muscles. Targeted training in these areas may enhance sprint economy and improve overall 400‐m performance. This perspective aligns with recent discussions in sports medicine, which emphasize the need for tailored training programmes that focus on the morphological characteristics of specific athletic events. The potential impact of these findings is significant, as they provide a basis for refining training strategies to enhance 400‐m sprint performance.

## Conflicts of Interest

The authors declare no conflicts of interest.

## Data Availability

The data that support the findings of this study are available on request from the corresponding author. The data are not publicly available due to privacy or ethical restrictions.
